# Simultaneous radio-ulnar cannulation: shifting gears from ‘feasibility’ to ‘applicability’

**DOI:** 10.1186/s43044-021-00229-7

**Published:** 2021-11-12

**Authors:** Ankit Kumar Sahu, Sudesh Prajapati, Danish Hasan Kazmi

**Affiliations:** 1 Department of Cardiology, Sanjay Gandhi Postgraduate Institute of Medical Sciences (SGPGIMS), Lucknow, Uttar Pradesh 226014 India; 2 Interventional Cardiology, Medanta Heart Institute, Amar Shaheed Path, Golf City, Pocket - 1, Sector A, Lucknow, Uttar Pradesh 226030 India

**Keywords:** Dual arterial cannulation, Peripheral vascular complications, Limb ischemia, Limited vascular access

## Abstract

Conventionally, routes of vascular access commonly include femoral and radial arteries with brachial, ulnar and subclavian arteries being rarely used for coronary interventions. Non-femoral arterial access is being increasingly preferred to minimise groin puncture site complications, prolonged immobilization and duration of hospital stay. However, radial artery cannulation is also fraught with fears of tortuosity, loops, vascular spasm, perforation, pseudoaneurysm formation, arm hematoma and arterial occlusion. In contemporary practice when most of the coronary procedures are being done via transradial access, encountering one of the above-mentioned hurdles often forces the operator to switchover to femoral access. Here, we explore the rationale, feasibility, operational logistics, clinical implications and future directions for using simultaneous radio-ulnar arterial access in the same extremity.

## Background

Simultaneous cannulation of both major arteries of the hand has always been feared with risk of breaching limb perfusion to the extent of need for possible limb amputation in future. This situation may arise subject to permutation and combination of various conditions for example, if radial access cannot be used because of anatomical barriers, when dual arterial access is required for simultaneous injections in CTO (chronic total occlusion) intervention, when the presence of severe peripheral (ilio-femoral) arterial disease precludes the use of femoral arteries, if vascular closure devices have recently been applied to bilateral femoral arteriotomy sites, when contralateral arm’s radial artery has been used up for bypass grafting, when femoral access has been utilized for intra-aortic balloon pump counterpulsation, ventricular assist device or ECMO (extracorporeal membrane oxygenation), if the patient is a multiple limb amputee or if patient and/or operator chooses to avoid femoral crossover access.

## Methods

**Fig. 1:**
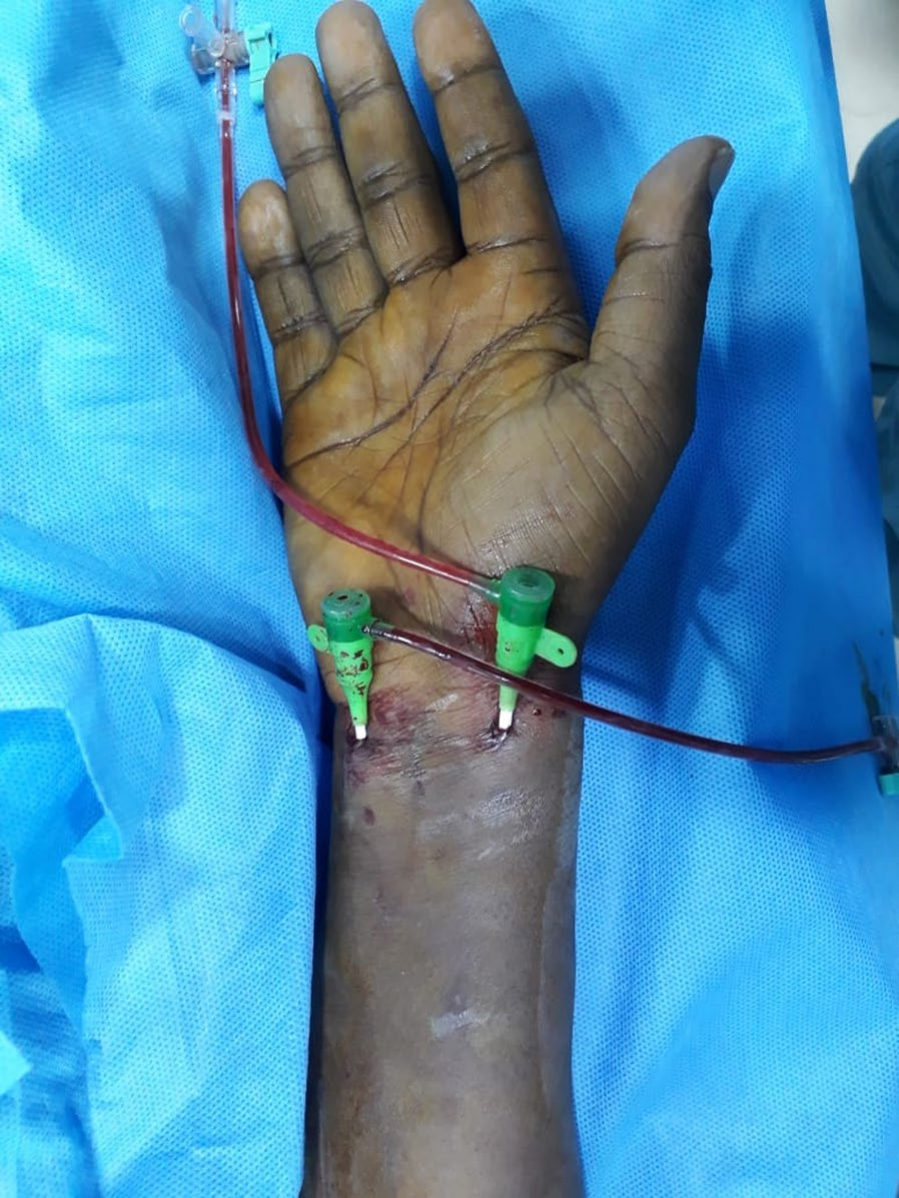
Concurrent cannulation of right radial and ulnar artery in a patient undergoing coronary intervention using 16-cm-long 6F hydrophilic coated introducer sheaths in right radial and ulnar artery (Glidesheath™, Terumo medical corp, NJ, USA)

We too encountered one such situation demanding single-arm, bi-arterial cannulation while performing left anterior descending CTO-PCI (percutaneous coronary intervention). The left radial was not patent due to a previous procedure, and right femoral artery access was precluded by a recent suture-based arteriotomy closure (Perclose Proglide™, Abbott cardiovascular, Plymouth, MN, USA). Right radial access was obtained for providing antegrade injections by engaging left coronary artery via guiding catheter while right ulnar access was used for giving contralateral injections by cannulating feeder vessel i.e., right coronary artery via diagnostic catheter (Fig. 1). Optimal inta- and post-procedure anticoagulation was maintained (ACT: 200–250 s). Sheaths were removed maintaining patent hemostasis ensuring that thumb and little finger remain perfused via digital non-invasive pulse oximetry after giving vasodilators per-sheath and infiltrating local anaesthesia. Trans-radial band was also removed after 1-h maintaining minimal local compression. Arterial patency was documented by vascular doppler ultrasonography prior to discharge and 1-year follow-up.

## Discussion

Although obtaining non-femoral same-limb dual-arterial access had been reported by Biondi-Zoccai et al. [1] in 2011 yet until recently there were only few sparse mentions of it scattered in the literature. Recently Kumar et al. [2] documented their experience, where none of the 16 patients with single extremity dual arterial cannulation for cardiac catheterization lost their arterial patency at 1 week follow-up. However, as described in their article and also in our experience, this fear of losing limb perfusion altogether can be allayed by taking precaution in the form of selecting patients with adequate palmer collateral network, screening for peripheral arterial insufficiency, avoiding arterial dissection while obtaining wire access, optimal use of peri-procedural anti-thrombotic therapy, reducing procedure time, proper and frequent flushing of sheaths, early sheath removal using patent haemostasis technique and using smaller, hydrophilic coated, tapered-tip introducer sheaths maintaining optimal sheath-to-artery ratio e.g., Glidesheath slender® (Terumo medical corp, New Jersey, USA).

Safety and feasibility of obtaining a homolateral ulnar approach in cases of radial approach failure have already been demonstrated in a prospective multicentre European registry wherein same-limb ulnar access was obtained successfully in 85.7% of patients having failed radial cannulation without any evidence of early limb ischemia [3]. Another modification which can be employed to avoid risking arm ischemia is to cannulate distal radial artery instead of proximal main radial artery as it prevents proximal arterial occlusion and does not jeopardizes hand circulation with simultaneous ulnar artery access ensuring successful passage as it is of relatively larger bore, less tortuous, devoid of loops and has relatively straighter course less liable for arterial spasm [4–8]. Vascular access-site complications viz. spasm, tortuosity, perforations, pseudoaneurysms and functional occlusions need expertise of competent invasive vascular specialists, if encountered while obtaining same-limb dual-arterial access.

## Conclusions

Concurrent radio-ulnar cannulation is often a result of necessity and lack of options rather than a matter of an elective choice. Clinical data although in the form of small pilot studies confirm the feasibility and safety of the procedure. Therefore, with proper indication, careful selection of patients, advent of advanced, atraumatic, vessel-friendly hardware and exercising due diligence and caution, successful simultaneous radio-ulnar access can be used for coronary interventions while minimizing complications.

## Data Availability

Not applicable.
